# Supplementary material for: plastiC: A pipeline for recovery and characterization of plastid genomes from metagenomic datasets
Source: Wellcome Open Res. 2024 Nov 27;8:475. Originally published 2023 Oct 18. [Version 2] doi: 10.12688/wellcomeopenres.19589.2 (PMC12498322; doi:10.12688/wellcomeopenres.19589.2)
Supplement: Peer review history

# Reviewer report: Reviewer response for version 2

**DOI:** 10.21956/wellcomeopenres.25383.r119188

**Published:** 2025-04-30

**Authors:** Anna Karnkowska, Michał Karlicki

**Affiliations:** 1 Institute of Evolutionary Biology, Faculty of Biology, University of Warsaw, Warsaw, Poland

**Review of *plastiC: A pipeline for recovery and characterization of plastid genomes from metagenomic datasets***

 The development of plastiC is a timely and valuable addition to the existing toolkit for analysing metagenomic data. Given the increasing interest in mining eukaryotic genomes, including organellar genomes, from huge metagenomic datasets, this tool has the potential to fill an important gap. However, we have some comments that could help refine the pipeline, increase its robustness and make it more accessible to a wider range of users.

**Major Comments:**

 1. The authors should discuss how plastiC compares to other methods for obtaining plastid genomes, such as the Tiara pipeline ( Tiara) and recent research on plastid genomes from TARA Oceans data (Jamy et al., 2025). Positioning plastiC in comparison to these methods would highlight its advantages and limitations.

 2. While plastiC is specifically focused on the recovery of plastid genomes, mitochondrial genomes are even more widespread. A brief discussion of whether there are similar tools or approaches for recovering mitochondrial genomes would provide useful context. Is there any particular reason not to use the pipeline for mitochondrial genomes (other than the fact that there is currently no module to assess the completeness of mitochondrial genomes)?

 3. The completeness assessment module is one of plastiC's greatest strengths. The approach of using KEGG and machine learning for completeness estimation is sound, but there are already similar methods, such as CheckM2. Could CheckM2 be adapted for plastid genomes and how would its performance compare? The novelty of the plastiC approach should be clearly emphasised. Also, completeness assessment often relies on detecting the presence or absence of predefined single-copy genes (see Jamy et al. 2025) - is the plastiC method more accurate than these approaches?

 4. The manuscript does not address the assessment of contamination, which is crucial when analysing complex environmental samples. Can contamination detection be integrated into the pipeline? If not, how does plastiC deal with cases where plastid metagenome-assembled genomes (MAGs) contain fragments of multiple plastid genomes or mitochondrial sequences? Could this lead to overestimated completeness?

 5. The dataset used to test plastiC appears relatively simple compared to typical metagenomes. The use of an aquatic environmental dataset containing plastid genomes from closely and distantly related species, mitochondrial genomes, nuclear genomes and bacterial genomes would allow a more realistic validation. Alternatively, a defined custom made dataset with known genomic fractions would also be useful.

**Specific comments**

***Introduction***

 1. The sentence "Plastid (or chloroplast) genomes are of particular interest because of their roles in primary production" should be revised. The genomes themselves are not directly involved in primary production, but the organisms that contain them are.

***Methods***

 1. The authors should specify the Tiara parameters used in the initial screening and MetaBAT2 binning process. Can these parameters be changed? What are the recommended probability thresholds and minimum lengths of the contigs for the different data sets?

 2. It is unclear whether the protein sequences in the training dataset were extracted with Prodigal or whether existing annotations were used.

 3. The exclusion of plastid genomes smaller than 94 kb (e.g. *Ostreococcus tauri*) raises concerns that completeness may be overestimated. The impact of excluding smaller genomes from the training set should be discussed.

***Results & Discussion***

 1. How do introns in some plastid genomes (which are highly expanded in certain groups) affect the tool? Was this taken into account in the training, test and validation datasets?

 2. Has the tool been tested on leucoplasts or other atypical plastid types?

 3. Can the completeness estimation module be used independently in other pipelines?

 4. In the case study, 13 lichen metagenomes were used, and although plastid contigs were identified in all cases, MetaBAT2 was not always able to generate correct bins. This bottleneck should be addressed. Could more advanced binning methods, such as DASTOOL, improve plastid genomes? The limitations of the approach should be better outlined.

 Overall, plastiC is a promising tool for the extraction of plastid genomes from metagenomics datasets. However, further validation on more complex datasets and a more detailed comparison with existing approaches would significantly extend the study. Addressing completeness estimation and contamination detection issues will further improve the robustness and applicability of this pipeline.

Are the conclusions about the tool and its performance adequately supported by the findings presented in the article?

Partly

Is the rationale for developing the new software tool clearly explained?

Yes

Is the description of the software tool technically sound?

Yes

Are sufficient details of the code, methods and analysis (if applicable) provided to allow replication of the software development and its use by others?

Yes

Is sufficient information provided to allow interpretation of the expected output datasets and any results generated using the tool?

Yes

Reviewer Expertise:

Microbial eukaryotes biology and genomics, evolution of organelles and their genomes.

We confirm that we have read this submission and believe that we have an appropriate level of expertise to confirm that it is of an acceptable scientific standard, however we have significant reservations, as outlined above.

## References

[1] : New deep-branching environmental plastid genomes on the algal tree of life. *bioRxiv* .2025; 10.1101/2025.01.16.633336 10.1101/2025.01.16.633336

[2] Tiara. Reference source

# Reviewer report: Reviewer response for version 1

**DOI:** 10.21956/wellcomeopenres.21699.r77563

**Published:** 2024-05-16

**Authors:** Yang Ni

**Affiliations:** 1 Chinese Academy of Medical Sciences and Peking Union Medical College, Beijing, China

Total comment: The development of the plastiC workflow marks a significant advance in the field of metagenomics, particularly with respect to the analysis and identification of plastid genomes from metagenomic datasets. This tool addresses a critical gap in existing metagenomic workflows, which predominantly focus on prokaryotic and eukaryotic genomes but often overlook plastid genomes despite their essential role in understanding the diversity and evolutionary processes of photosynthetic communities. However, several concerns need to be addressed to enhance the tool’s functionality and user experience:

  

1. Installation Challenges: There have been difficulties in installing plastiC following the instructions provided on the GitHub page. This issue may deter users and limit the tool's accessibility. A more streamlined installation process, potentially with a detailed step-by-step guide or troubleshooting section, could mitigate these challenges.
2. Standardization of Genome Assembly Evaluation: The current method for evaluating genome assemblies within plastiC does not appear to adhere to a standardized benchmark, which could affect the reliability of the results. It may be beneficial to reference established methodologies, such as those outlined in the paper "A Systematic Comparison of Chloroplast Genome Assembly Tools," to enhance the credibility and standardization of the assembly evaluation process.
3. Pipeline Workflow Optimization: The workflow of plastiC could benefit from further optimization. A clearly defined and efficient pipeline, possibly with enhanced automation and user-friendly documentation, would improve the usability of the tool. Additionally, incorporating visual workflow diagrams could aid users in understanding and navigating the process more effectively.

Are the conclusions about the tool and its performance adequately supported by the findings presented in the article?

Yes

Is the rationale for developing the new software tool clearly explained?

Partly

Is the description of the software tool technically sound?

Yes

Are sufficient details of the code, methods and analysis (if applicable) provided to allow replication of the software development and its use by others?

Yes

Is sufficient information provided to allow interpretation of the expected output datasets and any results generated using the tool?

Partly

Reviewer Expertise:

1. Plant mitochondrial genomics (major) 2. Plant chloroplast genomics 3. Plant Organelle genome data analysis tools/methods /pipeline 4. Anthocyanin biosynthesis metabolic pathways 5. Transcriptomics/multi-omics analysis

I confirm that I have read this submission and believe that I have an appropriate level of expertise to confirm that it is of an acceptable scientific standard, however I have significant reservations, as outlined above.

# Response

**Published:** 2024-11-27

**Authors:** Ellen Cameron

We would like to thank the reviewer for going through the manuscript and providing suggestions to improve the manuscript and our tool to increase accessibility and ease of use among users. 

 [1] We have revised our workflow such that the pipeline dependencies and tools are now stored in a Docker repository and the pipeline can be launched with the *–use_singularity­ *flag in Snakemake to automatically retrieve them. We believe that this will cut down on the amount of time and any challenges associated with the initial construction of the conda environments by the workflow. We have also added further detail to the README highlighting the steps of the pipeline, required input files and directory structures, and how to execute the pipeline.

 [2] *plastiC *does not serve to perform *de novo* or reference-based assembly of chloroplast genomes and utilizes pre-existing metagenomic assemblies for recovery, identification and characterization of chloroplast genomes. In “A systematic comparison of chloroplast genome assembly tools”, authors compare the quality of assemblies based on alignment of assembled chloroplast genomes to reference genomes. However, in the case of metagenomic studies, recovered chloroplast genomes may not have an appropriate reference genome available. We believe that by using the presence of KEGG orthologs, we instead provide a reference-free quality estimate of genomes inspired by the metagenomic tool, CheckM2, to circumvent these challenges.

 [3] We have added additional text description and figures to the GitHub repository to provide users with further context on the tool. We have also included visual examples of directory structures for input files to clarify how the input data must be organized for use in the pipeline.  Instructions for running and setting up the tool, including installation of Snakemake in a conda environment, is also outlined in the GitHub repository.

# Reviewer report: Reviewer response for version 1

**DOI:** 10.21956/wellcomeopenres.21699.r69060

**Published:** 2023-11-22

**Authors:** Adriana Lopes dos Santos

**Affiliations:** 1 Section for Aquatic Biology and Toxicology, Department of Biosciences, Universitetet i Oslo, Oslo, Oslo, Norway

The tool developed by Cameron and colleagues will be very useful to those working on advancing our understanding of the diversity and evolution of photosynthetic organisms. Chloroplast genomes or plastomes are often used to infer deep-level phylogenetic relationships among photosynthetic organisms, from single cell eukaryotes to plants. It also used to clarify phylogenetic issues arising from the analysis of a single or few marker genes. Comparative analysis of organelle genomes has allowed us to gain insights into ancestral architectures and evolution of these genomes. 

 Although chloroplast genomes can be easily sequenced from strains, the bulk diversity of photosynthetic single eukaryotes remains untapped. plastiC seems to offer the opportunity to explore the diversity of plastomes from uncultured organisms. In recent years, metagenomes have become the method of choice for investigating the composition and gene repertoire of microbial communities. The number of environmental datasets published has increased very rapidly, representing an incredible opportunity for data mining. However, several tools are orientated to the study of prokaryotic (bacteria) fraction of these datasets and a few to eukaryotic genes and genomes. 

 My only concern comes from the performance of the tool. The metagenomes chosen for demonstrating the effectiveness of plastiC were in someway simple in diversity. How the tool would perform in complex metagenome datasets such as those from marine samples, for example, where an array of photosynthetic taxa is expected to be found? 

 Also, I was wondering why the authors did not compare the recovered plastic bins with the available chloroplast genome from *Trebouxia* sp.

Are the conclusions about the tool and its performance adequately supported by the findings presented in the article?

Yes

Is the rationale for developing the new software tool clearly explained?

Yes

Is the description of the software tool technically sound?

Yes

Are sufficient details of the code, methods and analysis (if applicable) provided to allow replication of the software development and its use by others?

Yes

Is sufficient information provided to allow interpretation of the expected output datasets and any results generated using the tool?

Yes

Reviewer Expertise:

protist diversity, algae evolution

I confirm that I have read this submission and believe that I have an appropriate level of expertise to confirm that it is of an acceptable scientific standard.

# Response

**Published:** 2024-11-27

**Authors:** Ellen Cameron

We are pleased that the reviewer sees the value in the tool for exploring the diversity and evolution of photosynthetic organisms in metagenomic datasets.  We agree that lichens are a simplistic form of a metagenome but in the application here it was useful due to the expected abundance and signal arising from the photobiont. Technically, there are no theoretical limitations to the complexity of the dataset and the functionality of *plastiC*, providing the metagenome can be assembled. To further demonstrate the quality of genomes we recovered using *plastiC*, we have mapped the genome to a reference *Trebouxia *chloroplast genome (NC_045839.1). While this reference arises from a lichen photobiont, the exact species may differ and impact the mapping quality. These scores are now reported alongside other metrics of the recovered chloroplast bin.
